# Strengthening health systems and peacebuilding through women’s leadership: a qualitative study

**DOI:** 10.1186/s12992-023-00920-1

**Published:** 2023-03-23

**Authors:** Kristen Meagher, Mouna Khaity, Sali Hafez, Mariana Rodo, Nassim El Achi, Preeti Patel

**Affiliations:** 1 Research for Health Systems Strengthening in Syria (R4HSSS) and the Conflict and Health Research Centre CCRC, Department of War Studies, King’s College London, London, UK; 2 Independent Research Consultant, London, UK; 3 Department of Public Health and Policy, London School of Hygiene and Tropical Medicine, London, UK

**Keywords:** Health systems, Peacebuilding, Conflict, Women’s leadership

## Abstract

**Background:**

Active and protracted conflict settings demonstrate the need to prioritise the peace through health agenda. This can be achieved by reorienting attention toward gender diverse leadership and more effective governance within health systems. This approach may enable women to have a greater voice in the decision-making of health and social interventions, thereby enabling the community led and context specific knowledge required to address the root causes of persistent inequalities and inequities in systems and societies.

**Methods:**

We conducted a qualitative study, which included semi-structured interviews with 25 key informants, two focus group discussions and one workshop with humanitarian workers in local and international non-governmental organisations (NGOs), United Nations (UN) agencies, health practitioners, and academics, from Sub-Saharan Africa, Middle East and North Africa (MENA), and Latin America. Findings were then applied to the peacebuilding pyramid designed by John Paul Lederach which provides a practical framework for mediation and conflict resolution in several conflict-affected settings. The purpose of the framework was to propose an adapted conceptualisation of leadership to include women’s leadership in the health system and be more applicable in protracted conflict settings.

**Results:**

Five interrelated themes emerged. First, perceptions of terms such as gender equality, equity, mainstreaming, and leadership varied across participants and contexts. Second, armed conflict is both a barrier and an enabler for advancing women’s leadership in health systems. Third, health systems themselves are critical in advancing the nexus between women’s leadership, health systems and peacebuilding. Fourth, across all contexts we found strong evidence of an instrumental relationship between women’s leadership in health systems in conflict-affected settings and peacebuilding. Lastly, the role of donors emerged as a critical obstacle to advance women’s leadership.

**Conclusion:**

Continuing to empower women against social, cultural, and institutional barriers is crucial, as the emerging correlation between women’s leadership, health systems, and peacebuilding is essential for long-term stability, the right to health, and health system responsiveness.

**Supplementary Information:**

The online version contains supplementary material available at 10.1186/s12992-023-00920-1.

## Key messages

- Accelerating women’s leadership within health systems and peacebuilding is essential for long-term stability, the right to health, and health system responsiveness.
- Women hold a unique position in health systems as they facilitate more inclusive and improved service provision, well beyond sexual and reproductive health
- Though many peacebuilding projects are subject to political manipulation, the health system could be an entry point to mediate political differences within fragmented lines of control in complex conflicts.
- Women in conflict-affected settings face systematic and structural barriers in advancing to leadership positions in health systems and are often perceived as less capable of decision-making.
- There is limited donor and local funding and policies at the intersection of women’s leadership, health, and peace.

## Background

Since the 1990s, the World Health Organisation (WHO) and multilateral organisations realised the importance of health as a tool to pursue peace in conflict. In 1998, the WHO adopted “Health as a Bridge for Peace”, a policy framework with the premise that healthcare workers and the delivery of essential health programmes would contribute to the sustainability of peacebuilding in conflict-affected contexts such as Croatia, Bosnia and Herzegovina, and Myanmar [1–5]. Recent studies, however, demonstrate that the peace through health paradigm has largely been ignored by health professionals, policy makers and researchers working on health systems strengthening/rebuilding in conflict. A further core component yet to be fully explored is the role of women’s leadership in health in such contexts as it has shown to be of great added value in times of peace [6].

### UN initiatives on health, peace, and/or gender

There are three main UN initiatives that incorporate the three core elements of the study herein: the Women, Peace, and Security agenda (WPS), the humanitarian-development-peace (HDP) nexus, and the Global Health for Peace Initiative (GHPI).

The WPS agenda, implemented by the UN Security Council’s landmark resolution 1325, aims to increase the full and meaningful participation of women in conflict prevention and peacebuilding efforts [7]. The WPS agenda and their associated country action plans and policies focus on the participation and protection of women across sectors, not only in peacebuilding process. To date, 104 UN member states have adopted a 1325 National Action Plan, including Cameroon (2017), South Sudan (2015), Iraq (2014), Yemen (2019), Lebanon (2019), Sudan (2020). Despite the global momentum on the WPS agenda, its local and national manifestation is largely limited due to the voluntary national country action plans that are determined by countries priorities to do so [8]. For example, Afghanistan’s National Action Plan (NAP) was considered a critical milestone in advancing women empowerment and gender equality across sectors. The ambitious plan envisaged to work on encouraging women’s active participation in the civil society and political sphere through encouraging their participation in decision making process [9]. Consequently, that provided a roadmap to empowering women’s representation in senior leadership positions across sectors including health. The Taliban’s return to power in late 2021 has, however, reversed many of the gains made in women’s empowerment in the 20 years prior [10].

The humanitarian-development-peace (HDP) or ‘triple nexus’ concept dates from the twin resolutions on Sustaining Peace in the UN Security Council and General Assembly and the Secretary General’s inaugural speech in 2016 [11–13]. They both emphasised the need for development, peace and security, and human rights pillars to work together to prioritise prevention, address root causes and support institutions for sustainable peace and development to coherently address vulnerabilities before, during and after crises [11]. The humanitarian system is limited by its short-term focus, whereas development projects are focused on the long-term. Little is done from both entities regarding addressing and mitigating the drivers of crises and violent conflict. The nexus promotes improved coordination and cooperation mechanisms between different stakeholders for an effective transformation of crises into sustainable peace. The implementation of HDP faces multiple challenges that includes the limited understanding of the work and functioning of the other stakeholder groups, lack of incentive structures to encourage cooperation, and lack of joint analysis and scenario planning among the stakeholders. This is aggravated by the fact that the nexus has a broad concept which leaves significant room for interpretation and implementation from stakeholders [12, 14].

GPHI is a continuation of the Peace through Health policy framework. The latter is closely aligned with the UNSC 2282 (2016) ‘sustaining peace’ resolution, WHO’s Thirteenth General Programme of Work (2019 – 2023) which aims to ‘identify, harmonise and systematise its contributions to sustaining peace in fragile-, conflict- and violence-affected settings’ [15, 16]. GPHI aims to position health as an influencer of peace through health interventions by contributing to peace outcomes while pursuing health objectives [17]. This approach has already been successfully applied in multiple countries including Colombia, Somalia, Cameroon, and Burkina Faso [18].

### Health systems frameworks, leadership, and gender

WHO defines health systems strengthening as “any array of initiatives that improves one or more of the functions of the health systems and that leads to better health through improvements in access, coverage, quality or efficiency” [19]. These functions, as defined by the WHO Building Blocks (2007) include: service delivery, health workforce, health information systems, access to essential medicines, financing, and leadership/governance [19]. Leadership within health systems strengthening is the least well understood of these functions. Leadership is coupled with governance and is defined as ensuring strategic policy frameworks exist and are combined with effective oversight, coalition building, regulation, attention to system-design and accountability [19]. Its importance is often overlooked as not directly impacting health outcomes and therefore less critical in its role in strengthening health systems. However, Witter et al. describe leadership and governance interventions as potentially enabling health systems strengthening, given its cross-cutting nature across health systems and health more broadly [20]. Yet leadership and governance in health systems is not considered to be heavily influenced by gender or social relations, unlike other parts of the health system, for example services [21].

The failure to engage gender in leadership as a key component of health systems strengthening research and within frameworks and models has been increasingly recognised and has become a nascent research area. Hay et al. systematically reviewed several influential health system models [22]. They deemed the Control Knobs, WHO Building Blocks (2007), and the Universal Coverage Cube as mechanistic in nature, lacking any understanding of how they interact with the social environment, and any guidance on gender responsiveness [22]. While they found that other models recognise that health systems are dynamic and complex, they too do not provide intersectional gender analysis to understand how gender bias and restrictive gender norms affect health systems [22]. Hay et al. determine that “these findings highlight a missed opportunity to engage health systems in gender transformative strategies to improve health at a population level” [22, 23]. Studies from the ReBUILD consortium and Dhatt et al. demonstrate that greater parity and gender responsive, transformative leadership and capacity development is central for effective governance in health systems strengthening, improving service quality, and meeting global gender and health related goals [20, 24, 25].

Two gender frameworks specific to health systems research have been developed. Morgan et al.’s framework for gender analysis determines key domains that constitute gender power relations by asking who has what (access to resources); who does what (the division of labour and everyday practices); how values are defined (social norms, ideologies, beliefs, and perceptions), and who decides (rules and decision-making) [23]. Heise et al. present a conceptual framework for the gender system in health, which comprises five major pillars with direct and indirect interactions: (1) sex and biological determinants, (2) the gender system and the social production of gender (community context, political and legal frameworks, family influence), (3) gendered social positioning due to age, race, ethnicity, class, and ability, (4) different gendered pathways to health, such as exposures, behaviours, access to healthcare, and research, and (5) health inequities and outcomes [26]. In many settings, however, gender analysis including these frameworks, is not widely used [27, 28].

Health systems can therefore play a much greater role in the Health for Peace framework if we diversify thinking beyond traditional paradigms of health systems frameworks [6]. For example, public health measures, including equitable access to basic healthcare, may contribute to peacebuilding in the aftermath of conflict [29]. We can also go further than this, envisaging the conceptualisation of heath governance and leadership as a key contributor to peacebuilding in active conflict settings [30]. Our recent review of the north-west Syria and Afghanistan contexts supports the need to further explore the health for peace agenda by expanding traditional notions of leadership and governance within health systems, thereby going beyond the focus on health outcomes and amplifying the role of the systems themselves [30]. A purely health outcomes focus detracts from the importance of leadership diversity at the systems level. It completely disregards women as central to health systems strengthening from a leadership and governance perspective, focusing on women as primarily end-users and thereby reinforcing widely accepted gender norms and women’s subordinate role in influencing decision-making across society.

#### Protracted conflict

Conflicts are becoming more protracted, which requires innovative and long-term approaches taking into consideration the humanitarian development peace nexus and the role the health system has within this nexus [31]. Protracted conflicts are all-encompassing and key services, such as health, are often under-resourced and overwhelmed. Even where state systems remain effective, pockets of vulnerable areas can still suffer from violent conflict, splitting states, regions, and people onto opposite sides of a conflict, ultimately rendering any pre-conflict progress null and void [32]. Long-term strategies, rather than single interventions for acute conflicts, for example ceasefires for vaccination days, need to be employed [33]. Leadership in health in conflict can also be considered a long-term investment. A lack of investment in long-term strategies and interventions in conflict settings more broadly hinders the development of appropriate health systems strengthening agendas, as demonstrated in Syria and Afghanistan, where there has not been any sustained investment in the development of future women health systems leaders [34–37].

### Connecting the dots: women’s leadership, health systems, and peace

The evidence base exploring the connection between women’s leadership, health systems, and peacebuilding beyond narrative reviews does not yet exist [30]. There is growing evidence that links women’s leadership to health, primarily in post-conflict and stable high-income settings, and/or women’s leadership to peacebuilding [27, 38, 39]. Women are vastly underrepresented in leadership positions within health systems and in peacebuilding globally. Women make up more than 70 percent of the healthcare workforce, while holding only 25 percent of leadership positions [40]. Women from low-income and middle-income countries comprise just five percent of leadership positions in global health organisations [41]. While the statistics in conflict affected settings are largely non-existent, the wider trends do not bode well for such settings. In the last 25 years, just three percent of peace process mediators, witnesses, and signatories were women. Only two women have served as chief negotiators in major peace processes, including Stephanie Williams, acting head of the United Nations Support Mission in Libya, and Martha Karua in Kenya, and only one woman has signed a final peace accord as a chief negotiator Prof. Miriam Coronel-Ferrer in the Philippines [42–45].

Women are at the forefront of improving health for conflict-affected populations through service delivery, education, capacity strengthening, advocacy, and research [46]. Yet, women are also disproportionately affected by conflict and humanitarian emergencies [47]. It is well evidenced that, even when minor in numbers, women’s participation in peace negotiations with voice and influence leads to higher agreement implementation rates and longer lasting peace [38]. Since 2000, several international goals and resolutions have focussed on the role of women’s contribution and leadership in tackling pressing global problems, including the UN Sustainable Development Goals (SDGs) 3, 5, 10 and 16 and UNSC 1325 [7, 48]. Despite the evolving evidence base and rhetoric of increasing women’s leadership across health and humanitarian work, the status quo unfortunately persists.

Preliminary research findings demonstrate that this vital nexus may support the development of effective policies and interventions that adequately address the complexity and diversity of health in humanitarian crises and ultimately support peacebuilding [30, 42]. Due to multiple factors including the paucity of evidence, many public health professionals, policymakers, and researchers do not fully recognise or realise the linkages between health and peace. As a result, policymakers in fragile, conflict and violence-affected settings do not consider peace when developing and implementing health system interventions and health policies [49].

While leadership is often viewed as synonymous with a leadership position, in this study we define leadership as being recognised both formally (in a leadership position such as a Minister of Health) and informally (Community Health Workers who make meaningful decisions) in key decisions that strengthen systems, including health and/or create peaceful resolutions to conflict by working on a common vision. This definition builds on the peacebuilding and health systems literature, which envisages leadership across national, regional, operational, and local levels [50, 51].

In this pioneering study, we aim to explore qualitative evidence on the link between health systems, conflict, and peacebuilding through the role of women’s leadership. It is intended that this will contribute to organisational policy and practice to increase women’s meaningful participation in decision making processes in an array of organisations working at the nexus of health systems and conflict and ultimately support sustainable peacebuilding. The specific objective of our research was to undertake a field informed research project to identify how women’s meaningful participation in decision making processes at the nexus of health and armed conflict is supported or inhibited. The main research question was: *Can the advancement of women’s leadership at the nexus of health and peacebuilding offer a novel way of creating sustainable peacebuilding in conflict-affected settings?*In doing so, we focus on the various enablers and barriers to women’s leadership in health in conflict, its importance in peacebuilding, and ways to move forward and overcome multiple challenges presented in conflict-settings. We also explore whether Lederach’s framework of leadership, explained in more detail in the methods section, can be used for conflict settings or whether it needs tailoring to be fit for purpose.

## Methodology

### Theoretical framework

**Fig. 1:**
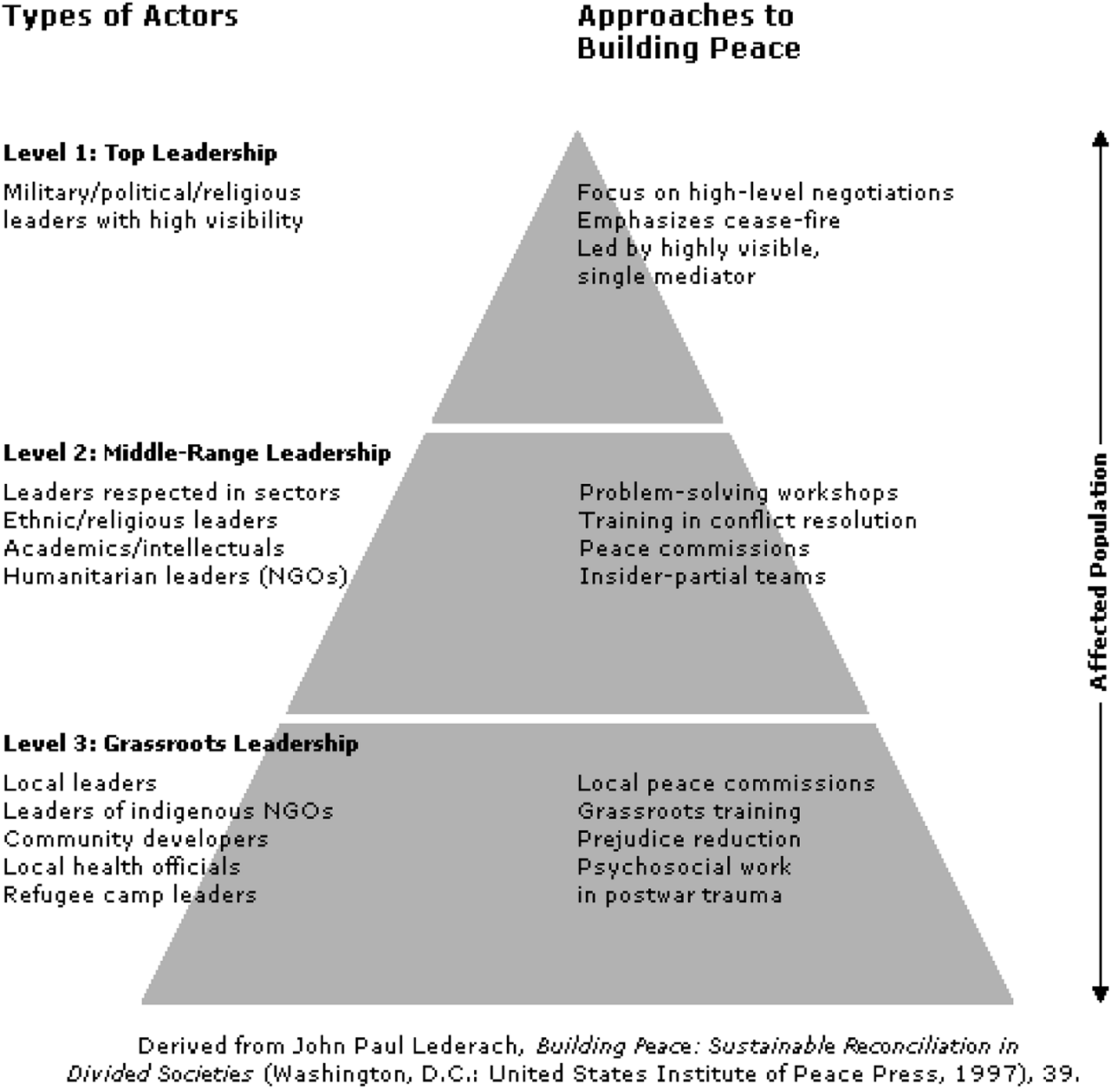
Peacebuilding pyramid

In this work, we adopted one of the widely used conflict-resolution, mediation and peacebuilding frameworks designed by John Paul Lederach [52]. It outlines Lederach’s central idea that there are three different levels of leadership involved in any conflict as well as the different approaches to peacebuilding appropriate at each level (Fig. 1). This is a particularly useful diagram for understanding the power dynamics between the three levels of the types of actors and the crucial role the middle-range leadership plays in ensuring access between grassroots and top-level leadership. Lederach is a proponent of building long-term commitment to “establishing an infrastructure across the levels of a society, an infrastructure that empowers the resources for reconciliation from within that society and maximises the contribution from outside" [52]. Furthermore, Lederach considers the cultural and contextual resources, both individuals and material, for peace as crucial in developing sustainable peace. This framework helped us in framing our analysis to better target the issue of leadership at the nexus of health and peacebuilding.

### Data collection

We employed a qualitative methodology, including semi-structured key informant interviews (KII), two focus group discussions (FGDs) and one workshop. The KIIs targeted primarily the research question and were used to generate key themes for analysis. The FGDs and workshop further corroborated findings from KIIs and provided recommendations.

We conducted 25 semi-structured key informant interviews with 18 women and 7 men, and two focus group discussions with 15 women and 3 men. The study took place between November 2021 and May 2022. KIIs were conducted remotely and FGDs took place in Gaziantep, Turkey. All KIIs and FGDs were audio recorded. To identify interviewees for KIIs, we used purposive sampling followed by snowball sampling; we recruited informants until achieving data saturation. We approached participants who are humanitarian workers in local and international NGOs, UN agencies, health practitioners and/or academics, from various levels of managerial positions. In addition, we included participants from various conflict-affected areas around the globe in order for this study to be a baseline for future in-depth context-specific research. Therefore, participants working in Afghanistan, Cameroon, Colombia, Egypt, Iraq, Lebanon, Libya, Somalia, South Sudan, Syria, Yemen, and Venezuela were contacted. Study participants characteristics are outlined in Table 1.

**Table 1:** Characteristics of key informants and focus group participants

Characteristics	National/Governmental (*N* = 5)	UN Agencies (*N* = 7)	International NGO (*N* = 13)	Local NGO (*N* = 16)	Academic (*N* = 2)	Totals (*N* = 43)
**Gender**						
Women	4	5	10	12	2	33
Men	1	2	3	4	0	10
**Nationality**						
National	4	4	9	16	1	34
International	1	3	4	0	1	9
**Managerial level**						
Senior management	1	5	8	6	1	21
Middle management	1	2	4	8	1	16
Field worker (health)	4	0	1	1	0	6
Field worker (peacebuilding)	0	0	0	0	0	0
**Country**						
Afghanistan	0	0	1	0	0	1
Cameroon	1	0	1	0	0	2
Colombia	0	1	1	1	0	3
Egypt	0	0	1	0	0	1
General	1	1	2	0	0	4
Iraq	0	1	0	0	1	2
Lebanon	0	1	0	0	0	1
Libya	0	0	1	0	0	1
Somalia	0	1	0	0	0	1
South Sudan	0	0	0	1	1	2
Syria	2	1	5	13	0	21
Venezuela	1	0	0	0	0	1
Yemen	1	2	0	0	0	3

To recruit KIIs for this study, we approached 69 potential KIIs who met the selection criteria specified above. five of them refused because they thought they were not the best person to be interviewed and recommended other people whom they considered to be more appropriate, 30 showed no response, and six responded after we had finished data collection, and three showed interest but did not respond to the request for scheduling the interview.

Researchers (MR, SH, MK, KM) conducted a total of 25 online KIIs between December 2021 and January 2022. Interviews were conducted via agreed online platforms and recorded when permitted (*n* = 25). The interviews were conducted in English (*n* = 13), Arabic (*n* = 8) and Spanish (*n* = 4). We developed an interview guide (Additional file 1) which was shared with participants ahead of interviews used to guide the process. The questions focused on the understanding of various gender and leadership related concepts, policies application in the organisation they worked for, and their views relating to women’s leadership and its relation to peacebuilding in the various contexts. The interviews lasted approximately 40–70 min.

Two FGDs were conducted by KM. Selection criteria for FGDs met the same criteria applied to the KIIs selection process and explicitly focused on the Syrian context. All participants were Syrian. The KIIs captured most of the information included in this study while the FGDs were intended to follow up on some of the main findings. The larger female FGD was to capture the nuanced relationship between women's leadership in the health system and peacebuilding. Including a larger more heterogenous group representing different sectors enabled us to explore the links between the complex topics studied here. Similarly, the female only FGD further ensured the quality and accuracy of the collected data as women may be reluctant to share their views and perceptions about gender relations if men are present [23]. The relatively fewer number of male participants in the first FGD may reflect the level of interest in the topic among men. The fewer members of the female participants of the second FGD may be influenced by the gendered roles in relation to work, household responsibilities, and family life, and as a result, this affected women's availability for attending the only- females workshop at short notice.

Additionally, we held one hybrid workshop at King’s College London with 13 participants, including academics and humanitarian workers in international NGOs. The purpose of the workshop was to seek feedback and corroborate our research findings. 

All data collected from KIIs and FGDs were transcribed using Otter.ai®, those in Arabic and Spanish were manually transcribed and then translated to English. All transcripts were anonymised by removing identifying information of the participants and using a unique identifier for each participant.

We used Dedoose-9.0.46® for data management and thematic analysis.

### Data analysis

For analysis, we adopted the six-phase process described by Braun and Clarke [53]. Firstly, we familiarised ourselves with the data by reading the transcripts. Secondly, each transcript was then coded by two researchers. Together, each pair of researchers discussed their coding approach to identify similarities and differences, thus avoiding interpretability bias. Thirdly, all researchers (KM, MR, SH, MK) shared the emerging codes and created a thematic framework for data analysis. Fourthly, the same researchers completed the open coding and started identifying emerging themes from KIIs and FGDs. Fifthly, all themes were defined and finalised. Finally, a completed narrative of the findings was written, supported with quotes. To further increase rigor, we focused on achieving both credibility and reflexivity. Regarding credibility, all KIIs and FGDs were transcribed verbatim, accurately translated into English where necessary, and utilised as the main data repository. As for reflexivity, and to limit biases, all team members were involved in the analysis and interpretation of results.

Ethical approval was granted by King's College London (LRS/DP-21/22–26,604). The information sheet and consent form were developed in English and translated into Arabic, Spanish and French.

## Results

Five interrelated themes emerged from our research. 1) The interpretations and perceptions of concepts including gender equality, equity, mainstreaming, and leadership, 2) the presence of armed conflict and its impact on the context, 3) women’s leadership in health systems, including barriers and enablers 4) the emerging relationship between women’s leadership, health systems, and peacebuilding, 5) donors approaches and policies, which highlighted external influences common in conflict settings. The connections between these themes suggest that there are a range of barriers and opportunities to advancing women’s leadership to strengthen the health for peace agenda which require further exploration through research, programmatic initiatives and strategies, and theoretical framing.

### Interpretations and perceptions of gender equality, equity, and mainstreaming

The perceptions and comprehension of concepts including gender equality, equity, mainstreaming, and leadership varied across participants and contexts**.**

*“The word ‘empowering’ may have 1000 interpretations.”—M, KII, Senior Management, National, Syria*

Few participants linked the misunderstanding that assumes a contradiction between gender equality and some religion’s interpretations and that the goal of gender equality is not to strengthen women against men and destroy society, but to achieve equity for both men and women to work and lead in their communities. Here, it is necessary that local communities are consulted in finding suitable definitions that are contextually appropriate and do not create hostile conditions that expose women to further vulnerabilities.

*“Men and women are different from birth, but this difference women only involves the genitalia […] all rights and duties must be equal for men and women[...] there is no difference between them, and Islam urges such equality.” -F, KII, Middle Management, national, Yemen*

For some participants, gender equality was defined as the affirmative action taken to promote equal representation, equal opportunities, flexibility, creating an equal environment for both men and women. While for other participants, including both men and women, gender equality is perceived as creating conditions and a work environment that go beyond fair representation, including creating policies that ensure positive discrimination for women in workplaces; greater understanding of gender equality within the health system, not only limited to access to health services and the burden of diseases from a gender dimension, but also achieving gender equality in promotion, employment and leadership representation, access to opportunities and information within the sector; adopting integrated gender equity across sectors; awareness of the challenges and problems that pursuing gender equality might create; ensuring equality amongst all people from different ethnic groups and genders within the same context; accountability of the power distribution and dynamics in society and including community participation in change, with attention to addressing the root causes that led to the existence of this problem; recognising and addressing toxic masculinity within the health system. Gender equality has also been linked to human rights, peace, and long-term development.

Perceptions about gender mainstreaming differs across contexts. Few participants expressed they are not familiar with the term and its meaning. We found a disparity in access to information about the term between local humanitarian workers and those working with UN organisations. Some participants defined gender mainstreaming as creating a work environment that ensures equal opportunities for women to participate in employment and decision-making.

*“We usually talk a lot about gender inequality. But gender mainstreaming […], ensuring […] that the definitions of gender and the understanding around gender issues is being streamed through different programmes, it doesn't have to be a certain programme or a certain intervention that talks around gender, but ensuring that gender understanding and definitions are being disseminated across different programmes in different sectors”-F, KII, Senior Management, International, Egypt*

Other participants defined it as a broad concept meaning inclusion of gender equality in all programs; awareness of how gender affects work and implementation plans and the need to include the gender lens in intervention’ planning, monitoring and evaluation, and providing gender-sensitive assessment tools for analysing indicators and data; rethinking of gender and sexual orientations in a specific humanitarian context; analysing all sources of violence, power relations, access to resources and challenges experienced by people along gender lines and including this analysis in the national development policy framework and strategies as an essential step for achieving gender equality and preventing discrimination.

*“By including […] gender, it is possible to raise the profile of other types of violations that historically have been hidden. So, being able to include a gender perspective will allow a broad, meaningful analysis which recognises the violations which the people have been exposed to..” - F, KII, Field worker: health, national, Colombia*

Gender, social, and cultural norms guide the perception of leadership across contexts and who is considered a leader, and what kind of decisions they can make. Women are largely still perceived as less capable to take political decisions for health.

*“I think the leader we see worldwide tends to be a more masculine form of leadership like this idea of the strong, uncompromising strong man, that's what power is about the sort of populace like culture, personality, unwilling to compromise. [...] I think there's a lot [who] suggested that model of leadership isn't what builds peaceful societies that peaceful societies are built on the willingness to listen, to be inclusive, to bring people and to admit when you're wrong, all of those kinds of things, which tend not to be associated with what strong leadership looks like” F, KII, Senior Management, international, Lebanon*

In a similar manner to that of defining equity, equality, and mainstreaming, note that very few of the participants were able to recognise and mention at least one of the three initiatives, WPS, GHPI, or HDP nexus. Those who did appeared to be more experienced in terms of applying these initiatives rather than having a deep understanding of the theory behind them.

### The dual role of armed conflict

Armed conflict affects women healthcare providers’ lives and career choices. We found that conflict itself is both a barrier and an enabler for women’s leadership in health. In Syria, the conflict reinforced prior gender inequities, including restrictions on movement and consequently women’s access to leadership and coordination positions in the humanitarian health response. While in Somalia, conflict facilitated women’s movement and hence women played a leading role in coordinating the humanitarian health response between the different areas of conflict.

*“In fragile contexts like Somalia, the NGOs or the health facilities that work in conflict zones tend to be mostly headed by women. The fact being that they are non-combatant they are not seen as a threat have better access, ability, or freedom to move across borders.”- M, KII, Middle Management, National, Somalia*

*“Security was, I believe the second factor after the social factor […] the social played together with the security to push women away from the leadership.” - M, KII, Senior Management, National, Syria*

Security concerns are a barrier that prevent women from accessing leadership positions or developing their skills and experiences, which leads to gendered access to information. Security concerns mentioned include fear of being kidnapped or rape or sexual abuse, or being targeted during military operations.

Political barriers in conflict settings also play an important role in restricting women’s access to leadership, where the rise of some political parties and de facto authorities led to the implementation of practices and policies aimed at excluding women from decision making. For example, separation of men and women in workplaces, preventing women’s movement without a male guardian, restricting women’s/feminist organisations, and limiting women’s roles to ineffective positions within the governance structures. These practices led to the exclusion of women from political spaces, interfering with health policies’ designing, and participating in health response decisions as in the case of COVID-19 responses in Yemen and Syria.

We did not systematically examine the traumatic situations and coping strategies to overcome the trauma that healthcare providers face during the conflict. However, the need for mental health support was discussed in several contexts, as a way for people to cope with traumatic experiences during armed conflict without resorting to familiar patterns of violence; addressing macroaggressions; and improve organisational culture.

*“[On ways to address obstacles to women’s leadership] are topics of psychosocial accompaniment to all men and all women. Because we have a life story, and that life story is what brings us...if we have not healed internal things it leads us to attack the other, [...] and this [psychosocial accompaniment] improves the organisational climate.”* – F, KII, *Senior Management, National*, Colombia

The need to provide mental health services with an intersectional feminist approach emerged to address the connections between the socio-economic and political and personal barriers, especially since women suffer from violence in these contexts two-fold – direct conflict or war violence and indirect patriarchal violence. Providing mental health services was seen as contributing to peacebuilding, by supporting people in decision-making, bringing perspectives together, and enhancing dialogue opportunities at the community level. In South Sudan, women adopt certain psychological support strategies, such as storytelling about the violence that women or their children face as a way to conflict prevention and resolution.

*“We know that part of this is the necessity of psychological support, and assistance in overcoming the trauma of war and of a patriarchal society, but we have not got much capacity here. We would like to make referrals to other institutions, but we feel the lack of psychological support in general, and the lack of a feminist approach, and a great deal of reluctance to deal with male and female survivors as survivors and not as victims. In other words, the feminist approach is an investment.”- F, KII, Senior Management, National, Syria*

### Women’s leadership in health systems

The health workforce in conflict settings, like other settings, reflects a strongly gendered pattern where women are clustered in mid and lower-level cadres, and health leadership is mostly occupied by men. This was echoed in our research findings, where the gendered division of labour emerged as a key barrier to leadership. In most settings, we found women face systematic and structural barriers to participating in and advancing leadership positions in the health system, similar to the wider literature on women’s leadership in health systems [54]. Advancing women’s leadership in conflict settings is exacerbated by security issues and systems wide patriarchal attitudes emboldened by the presence of conflict [47, 55, 56]. Lack of political will to enhance women’s leadership in health, alongside the policies adopted by de facto authorities in some contexts, contributed to reversing the privileges that women have recently achieved regarding access to leadership positions. For instance, in Afghanistan, the Taliban policy of gender separation of men and women systemically excludes women from entry, progress, leadership, and decision-making in health.

*“The systemic exclusion of women is politically driven. We heard in some provinces, issued regulations, strict rules for NGOs, they must have separate offices for female and male staff. And the women have to be covered even during office hours, and […] accompanied by the male family member who has to wait for them inside the office.” -M, KII, Middle Management, International, Afghanistan*

Our research found that addressing structural and systemic challenges contributes to retention and fostering women’s leadership in health systems in conflict within organisations. In Egypt, Syria, Libya and Yemen, the governance system of the public health system and/or parallel health system lacks the protection dimension that structurally offers favourable working environments for women. Lack of implementation of accountability mechanisms regarding sexual harassment was mentioned, alongside using sexual harassment as an exclusion practice that forces women to not seek health leadership positions. Human resources are also an area where INGOs and national and/or parallel health systems diverge, this creates additional disparities in enhancing women's leadership even in the same context. KII and FGD participants stated that Human Resources departments of INGO and UN agencies go beyond administrative issues and focus on protection and empowerment policies by developing and enforcing women’s empowerment policies, zero-tolerance policies, and ways to encourage women.

*“The HR department (in INGOs) cares about these issues and works to address these challenges. The public institutions with their unawareness of these issues results in them acting in this way, so if the public institutions would dedicate a department... in fact there is a department already for human resources we call it staff management department but they are very far from these policies that we see in international organisations..” F, KII, Middle Management, National, Libya*

Women also face organisational barriers in the public sector that fail to promote gender mainstreaming and women’s leadership. INGOs and UN agencies demonstrated gender-sensitive organisational culture and working environments in comparison with national organisations. In fact, in Libya and Yemen, the organisational culture of the public sector is deemed discouraging and disempowering to women. Gender discrimination policies in both governance and human resource structures, alongside the gendered access to information, including training opportunities were found to be key barriers to advancing women’s leadership.

*“When the Taliban came to power, the women were removed. So maybe they kept the title. But they put a man. […] Like, the woman knows that she can’t really open the mouth. If the male colleague say something, then she has to obey. So I think they should have they should put more women into [health] departments.” - M, KII, Middle Management, International, Afghanistan*

Social and individual barriers intersected with organisational and structural barriers to create additional obstacles for women’s leadership in the public sphere and within health systems. Across contexts, social norms, and cultures, patriarchy emerged as key challenge. The gendered division of labour within workplaces and households, caring responsibilities, and lack of family support were cascaded for understanding leadership in the health sector. Religious interpretations also emerged as a barrier to women’s workplace advancement. Framing women as beneficiaries and victims rather than leaders was also detrimental in advancing leadership capabilities.

*“The statements saying that women are incapable of leading rely on some religious interpretations which are fictional ones.” M, KII, Senior Management, international, Yemen*

*“There's this conception that women are not tough enough to take political decisions. [Men] think that we mix our emotions when [we make decisions]” F, KII, Field worker, National, Cameroon*

As a result of the barriers, women are underrepresented in leadership in all contexts in health systems. These barriers intersect and may create additional context- specific barriers. For example, in the Syrian context, the humanitarian response for the northwest is led by the WHO cluster in Gaziantep in Turkey. We found women are underrepresented in headquarters in health-based NGOs with limited access to contributing to political decisions for health. Women in northwest Syria face double discrimination in accessing health leadership positions on the ground, as well as the ability to influence the political decision for health led by health-based NGOs in Turkey. Thus, in this context, we find an additional obstacle to women’s leadership related to the geographical presence of women.

To overcome these barriers, women, as individuals and feminist NGOs, have adopted various coping mechanisms to push towards meaningful representation of women in leadership positions and implement gender-sensitive human resource policies. Solidarity, creating networks, long-term investment in women’s leadership, and volunteerism, outside and inside the workplace, was adopted by individuals, women’s organisations and coalitions to continue efforts in awareness raising and advocating for women at the local and international levels.

*“[Women] are not the leaders, they are not always the spokespeople, but the act of varying the voices helps. We have a significant process in […], we’re creating a network of female protection builders. And these women are doing an incredible job.” F, KII, Senior management, National, Colombia*

### Women’s leadership, health systems, and peacebuilding nexus

Across all contexts, we found strong evidence of an emerging instrumental relationship between women’s leadership, health systems, and peacebuilding in conflict-affected settings. Our findings demonstrate that there is an understated value in advancing women’s leadership in health systems, while implementing activities that actively expand the link with health and peace. This requires a multifaceted approach, in which women are provided with the opportunities to advance as health systems leaders while also being actively involved in developing peacebuilding skills.

*“The great status that female doctors enjoy enables them to participate in peacebuilding. The people who work with them feel very positive […] Therefore, if there is an effective peacebuilding training programme where female doctors are trained to be an integral part of it, in this way we will be hitting two birds with one stone.” – M, FGD, Senior Management, National, Syria*

At this nexus, health is framed as a sign of stability and a key pillar of establishing peacebuilding initiatives in conflict settings, as illustrated in Afghanistan, Yemen, Libya, Syria, and Iraq. The nexus highlights the right to health, as in the case of South Sudan, where war is framed as a public health issue. It furthermore shows how community health, in general, cannot be achieved without addressing all socioeconomic, gender, and political inequalities. We found evidence that health services are also linked to achieving stability and a sense of community belonging, since health broadly, ensures the involvement of all individuals on a large scale in developing coping mechanisms in conflict and reconstruction efforts in post-conflict.

*“These two things [health and peacebuilding] are tied together. If you are already a leader in health system, you should be a leader in peacebuilding. The only thing is that correlation has not been explored. But by design that correlation already exists […], at least in our context. Health workers are trusted.” M, KII, Middle management, national, South Sudan*

While health systems have been weaponised and subsequently politicised in various conflict contexts, participants stated the health system cannot be ideologically divided like other sectors [57, 58]. It is a common interest of all citizens to access health services regardless of political affiliation. Therefore, this nexus can be used as a tool to create a dialogue with communities as health professionals are trusted, highlighted in the cases of Syria and South Sudan. In South Sudan, one interviewee stated that stand alone peacebuilding projects are prone to political manipulation, therefore health can be used as entry point to peacebuilding given its relative neutrality. The presence of health coordinating bodies contributes to creating a common space for healthcare workers and leaders from different areas of influence in the same setting. Key informants and FGD participants emphasised how women play an important role in direct communication with communities and across different areas of influence, where women are considered peaceful, which ensures their freedom of movement, as in Somalia.

*“When a Health Partner goes out to deliver services, they can cross lines of battle and go and access communities from the other side […] even in the most difficult, the most polarised communities, you still have that opportunity to deliver services across these lines. That means you also have an opportunity to deliver peace across the same lines.” - M, KII, Middle management, national, South Sudan*

### Donors’ approaches and policies

Donors’ policies and approaches were well-examined in our research, given their influence in conflict-settings. Participants expressed a lack of connection between contexts and donors, explicitly noting that there is often significant disconnect and lack of engagement with local organisations, whereby donors might be engaged with INGOs but not directly local organisations which exacerbates the disconnect between the context and the donor.

Donors across contexts adopted the following approaches and practices:

- Scarce funding available for women’s leadership.
- Blueprinting across contexts without developing contextually relevant and tailor-made policies and projects.
- Lack of commitment to ensure sustainability of funding, which has a particularly negative impact on women and exposes them to greater vulnerability.
- Adopting protection policies that focus overwhelmingly on gender-based violence that beneficiaries, not healthcare providers, face.
- The competitive nature of funding means donors are often the ones setting the agenda for funding requirements. Local and international organisations, therefore, tailor projects to the needs of donors rather than local populations.
- Condoning organisational policies imposed by local partners that are discriminatory against women’s participation and leadership.

Funding programmes and organisations that support women’s leadership, education and capacity strengthening emerged as a key finding to empower women. This includes women-led civil society organisations, as well as national health systems, such as Ministries of Health and directorates of health to address the gap in women’s leadership and the limited gender responsiveness of health systems. For example, the partnership between a feminist organisation and the Idlib Health Directorate in north west Syria contributed to the co-design and co-delivery of a programme to increase the number of women in the health sector based on attention to the sensitivities of the local community. This further opened the door to discussions on the importance of the gender dimension in designing programmes aimed to strengthen the health system and build human health resources.

Participants emphasised the need for donors to engage with local women’s initiatives as this may support increasing the connection between donors and the realities on the ground. Furthermore, creating an accountability system to measure the real impact of women empowerment programmes, and shifting toward feminist intersectional funding approaches. Participants noted the importance of developing contextualised evidence-based research as a tool to advocate and turn research into policy, especially GBV within the health system.

Diplomatic leverage combined with affirmative action and advocacy efforts will ensure stronger women’s representation in health responses and health systems. In Afghanistan, there is an initiative to build an in-country advocacy coalition for a gender-responsive health system and to strengthen women’s leadership in health.

*“I think a large impact can be made particularly on the allocation of funds determined in Brussels for example, if women were present in senior official meetings, it would make a lot of difference.” Syria, FGD, Middle Management, F*

## Discussion

This is the first qualitative study to examine the link between women’s leadership in health systems and peacebuilding in multiple conflict and post-conflict settings. Indeed, previous studies have focused on one or two of the three components, but have never assessed the link between women’s leadership, health systems and peacebuilding, let alone in active conflicts as the literature focuses primarily on post-conflict and reconstruction contexts [30, 42, 59].

**Fig. 2:**
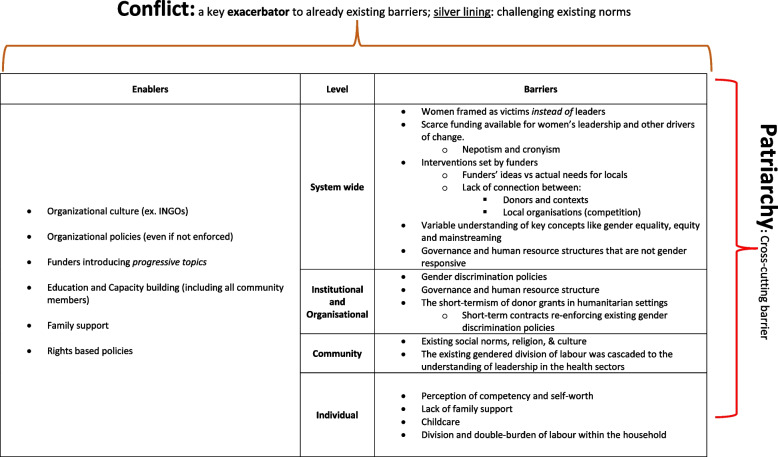
Barriers and enablers to women’s leadership at the nexus of health and peacebuilding conflict settings

We included voices of professionals from different levels of the workforce hierarchy, in local or international organisations, for the purpose of conveying the intersectional experience of individuals and organisations towards the barriers and enablers for women's leadership (Fig. 2). This has enabled a cross cutting and holistic understanding of the various factors, at various levels, that affect women’s potential for progression towards leadership in both health systems and peacebuilding.

Our findings are in accordance with the literature that highlights the barriers to or exclusion of women’s leadership in both health systems and peacebuilding initiatives [24, 27, 37, 60–62]. These barriers include prevailing patriarchal societal attitudes encompassing gender norms, biases, and assumptions that exclude women from areas of influence and power; shifting power dynamics with the presence of militarised and external actors; insecurity; sexual harassment; lack of capacity building and unequal access to resources; discriminatory organisational or donor related policies, all of which were also mentioned by our informants [24, 46, 63–66]. This demonstrates that such barriers are beyond a specific field, domain or context, and that they are exacerbated in conflict, thus requiring drastic changes to tackle their root causes at the societal, organisational, and system-wide levels, by implementing actionable policies [46, 47].

Given the fast-changing political situation, political coalitions, and that international recognition of de-facto authorities undermines the macro policy sphere in conflict settings, an all-encompassing, gender inclusive, health system may provide an opportunity for further exploring the link between peace and health in active conflict. Such a system may also support long-term investment in other systems impacted by conflict, as it is more inclusive and thus more perceptive to community wide needs [24, 42, 47]. This would transform the inadequacies of short-term focused humanitarian aid and promote a development mindset even in the early stages of conflict. Furthermore, this may support, international policy recommendations from bodies such as the UN, including the WPS agenda, the triple nexus, and the GHPI, to have more policy relevance and impact structural change at the community and individual level [30, 42].

This study, in line with previous studies focusing on post-conflict contexts and the emerging literature in active conflict settings, suggests multisectoral and organisational structural reform as a way to overcome barriers to women’s leadership [24, 42, 46, 67]. This would be through the implementation of national and organisational gender-sensitive policies, ensuring minimum representation in decision-making in organisations, including informal quotas and prioritising women for different opportunities, and education and capacity-building workshops to empower women and enhance their confidence and self-perception of how capable they are of advancing and seeking leadership positions [24, 27, 60, 68, 69]. However, uncertainty is the pervasive reality in protracted conflict settings with unpredictable political complexities which creates a governance void that, as in the case of northwest Syria, is filled by multiple international actors and donors rendering them key drivers for change [46, 56, 70]. It is therefore critical that international actors and donors address these necessary changes in cooperation with the needs of local actors in the active conflict phase to support long-term gender inclusive reform.

This study highlighted the dual role of conflict in both promoting and hindering women’s leadership in health and peacebuilding. For instance, though women are disproportionately impacted by conflict, with significant rise in gender-based violence (GBV), along with limited mobility, conflict creates a space to challenge gender norms and promote drastic societal changes in a (relatively) short period of time. This has already been indicated in our previous work and in multiple references in the literature [39, 46, 71]. This shows that countries affected by conflict are fertile grounds for change on the condition that other enabling factors are present. For example, it has been reported that in areas under the Assad regime, multiple social norms have been broken, in contrary to opposition-held areas where “the alliance of the various de facto forces with the traditional sectarian, tribal, family and regional institutions have led to a decline in the already weak role of women in contributing to decision-making at the regional level, confining most of them to housework” [72, 73].

The most significant finding from this study, however, is the instrumental relationship between women’s leadership in both health systems and peacebuilding in conflict-affected settings. We found this correlation is a key pillar for long-term stability and community belonging, creating dialogue within communities and across different areas of control in the same setting, establishing peacebuilding initiatives, the right to health, health system responsiveness, and conflict prevention. Indeed, there is widespread consensus among practitioners and scholars that peacebuilding can be more effective if built on an understanding of how gendered identities are constructed through societal power relations between and among women, men, girls, boys, and members of sexual/gender minorities [63, 74]. Findings from a study in Uganda linking education, gender and peace demonstrate gender equality and sustainability in peacebuilding through a country’s public institutions and social services − such as education and health − cannot be detached from how rigid gender roles and persistent power dynamics are culturally, socially, politically, and economically perpetuated and reproduced [63].

Similarly, the value of incorporating gender as an essential component of strengthening health systems in conflict is increasingly acknowledged [30]. Percival et al. states that the gender-blind nature of health system engagement has missed an important opportunity to contribute to more equitable and peaceful societies, given the frequent contact made by individuals with health services, and the important role of the health system within societies [75]. Women’s leadership in health systems in conflict creates a potential entry point for peacebuilding and can be considered as an overlapping theme which demonstrates a strong synergy between WPS, HDP, and GHPI [7]. For instance, in the HDP nexus, gender inequality is considered one of the root causes of the emergency/humanitarian needs and that proper transition to the development phase necessitates targeting gender inequality, along with other issues [76]. Similarly, the WPS agenda, calls for full, equal and meaningful participation of women in conflict prevention and peacebuilding efforts, with multiple studies highlighting the unique negotiation skills that women have [38, 42]. Moreover, and as explained earlier, Health for Peace highlights how health is an effective entry point for peacebuilding; though missing the women’s leadership element [6]. Therefore, promoting women’s leadership in health systems and peacebuilding in conflict settings can be viewed as remarkable policy panacea for these initiatives and a way forward to surpass some of the traditional paradigms of the WHO’s Health for Peace programme. Note that though highlighted in this work, there is still much to explore about the women’s leadership, health systems and peacebuilding nexus in general, and in conflict settings more specifically, given that conflict is a key driver of the dynamics between the entities involved in this nexus.

As conflicts become more protracted and that most of the world’s extreme poor could live in fragile, conflict and violence-affected settings by 2030, investment in research exploring this nexus has never been more critical [32]. This would ultimately support peace and stability at the community level and peacebuilding initiatives more widely. For this to be realised in practice, approaches to peacebuilding must include women’s leadership and health systems at all levels. We therefore argue that peacebuilding frameworks must be revised to include this.

### Lederach’s peacebuilding pyramid: a contextual adaptation

**Fig. 3:**
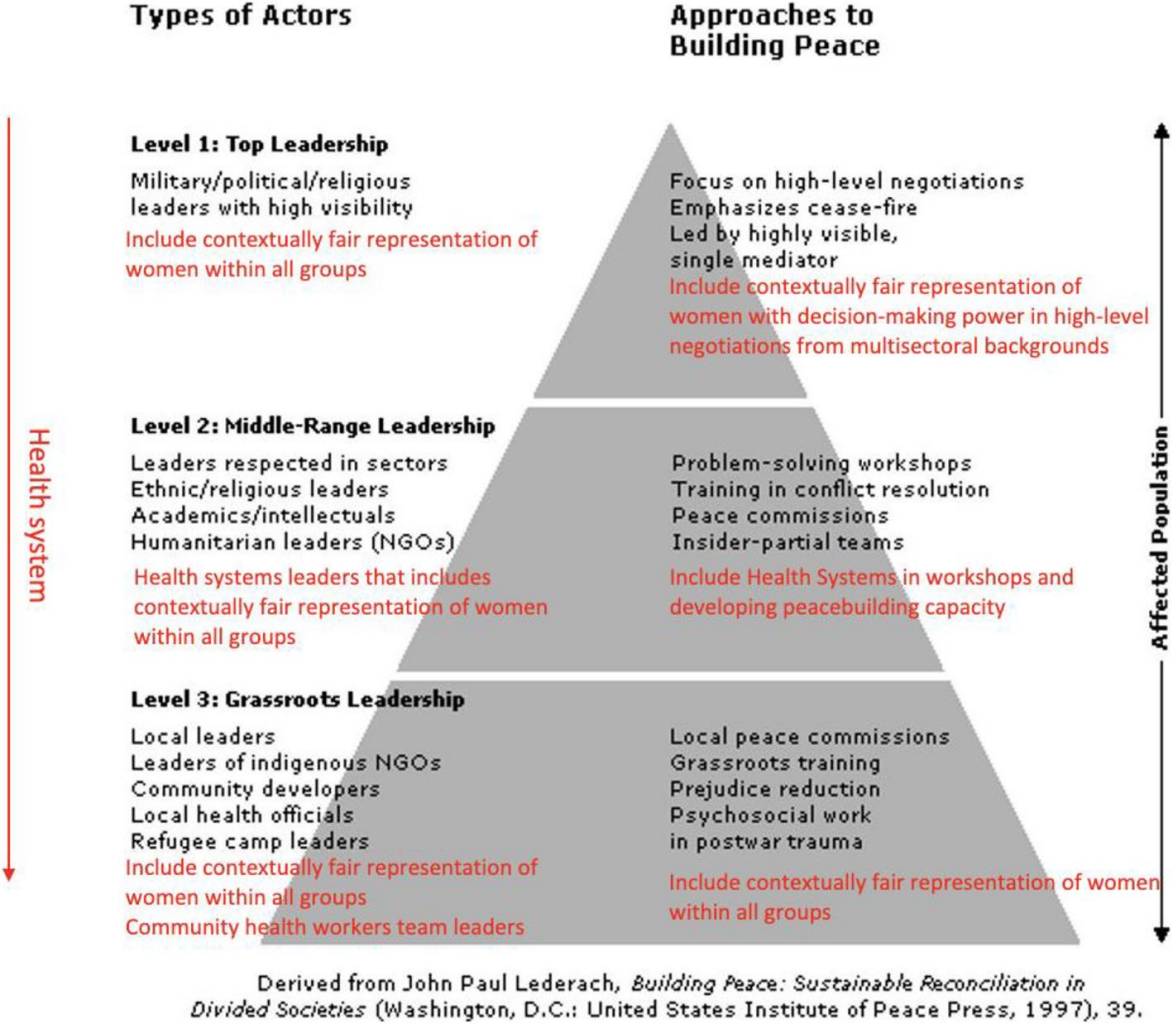
Adapted peacebuilding pyramid

To develop further understanding of how our research findings may support peacebuilding and challenge the overarching patriarchal systems that significantly influence where the power lies in decision-making, we have reviewed John Lederach's peacebuilding pyramid to see to what extent it can be adapted. Our findings show that health systems are relevant for peacebuilding but none of the health systems frameworks incorporate peace, nor do they focus on leadership the way that the Lederach framework does, and therefore we chose to build on this framework. The key themes that emerged from our findings reflect the complexities of addressing the inclusion of women’s leadership at the peace and health nexus, highlighting the structural and organisational barriers, as well as the array of stakeholders involved. Though Lederach’s framework extensively covers the complexity and changing dynamics between the various stakeholders to achieve peacebuilding, we argue it is necessary to develop this in an era of protracted conflicts by engaging other systems at a much deeper level, namely the health system, and incorporating a gender element as identified by the themes in our findings. It is crucial to establish intersectoral dialogue with gender focal points that have leadership and decision-making capacity to go beyond the current narrow focus and create more entry level opportunities for women. This will also engage local health systems strengthening initiatives and partnerships to develop institutional and individual capacity that goes beyond one sector. It may further support the development of improved health systems frameworks, acknowledging not only the fundamental role that women’s leadership plays in strengthening such systems, but also demonstrating that health systems frameworks are important in overcoming systematic and structural gender inequalities, not just improving health outcomes. Our proposed adaptation of the pyramid is indicated in Fig. 3.

**Table:** 

Finding	Recommendation
*For donors and policy makers*	
Limited funding and resources to develop	• Develop sustainable long-term, flexible funding streams on women’s leadership, health, and peace
Inadequate structures and systems to support women leadership in health and conflict	• Engage with local health systems strengthening initiatives and partnerships to develop institutional capacity • Utilise diplomatic leverage and affirmative action through policies to influence national or de-facto governments and organisations to ensure greater women’s representation in health and health systems at the decision-making level
Limited intentional programs, opportunities and funding for women leadership in health and conflict	• Introduce training programmes on microaggression, mentorship and shadowing, and feminist research design • Invest in future and emerging leaders in health, including women and gender minorities • Shift towards gender responsive budgets as a component of all development and humanitarian aid projects • Fund evidence-based research: gender disaggregated data and assessment of gender responsiveness (policies) of different national and de facto health systems
Limited space for discussion around women leadership in health and conflict and its reprioritisation	• Lead and support collaborative bilateral and multilateral networks aiming to advance women’s leadership in health in conflict
*For the research community*	
Limited literature and evidence on women leadership, health, and conflict, particularly those produced in the conflict affected areas	• Develop methodologies and tools for gender analysis, and standardised terminology to ensure a clear and consistent understanding and communication about gender • Fund initiatives that focus on leadership and decision-making at all levels in a multidisciplinary manner
Social and individual barriers intersected with organisational and structural barriers to create additional obstacles for women’s leadership in the public sphere and within health systems	• Fund activities and initiatives that focus not only on women’s empowerment but also work with men to address baseline perspectives, awareness raising of gender inequality and microaggressions
*For national and de-facto health systems*	
Disparity in access to information about the term between local humanitarian workers and those working with UN organisations	• Implement contextualised training of all health workers on key concepts and practices related to gender that influence the work environment • Promote system-wide gender mainstreaming to health and leadership across de facto, national, and international systems
Limited representation of women in decision making	• Support enabling and inclusive work environments not based on tokenistic quotas
Framing women as beneficiaries and victims rather than leaders was also detrimental in advancing leadership capabilities	• Adapt holistic, transformative, and rights-based approaches with gender sensitivity across all activities and system approaches • Establish intersectoral dialogue with gender focal points
*For the Women, Peace, and Security community*	
There is an understated value in advancing women’s leadership in health systems, while implementing activities that actively expand the link with health and peace. This requires a multifaceted approach	• Strongly consider the role of health workers and the health system in peace negotiations • Enhance multi-sectoral and multidisciplinary approaches to peacebuilding

### Limitations

This study has several limitations, largely relating to the limited time available to conduct this study. Firstly, we were unable to obtain interviews across a wider range of conflict-affected contexts, limiting our findings to a select number of countries. Secondly, while we have included professionals from different organisations, at various levels and local and international staff, there remains limitations analysing local or community level leadership and with national staff working in international organisations. Additionally, we did not compare women’s leadership for international staff versus women’s leadership for local staff. We also did not investigate sub-national disparities, especially in the countries that witness fragmentations in the leadership of the health sector and the political system, as in Yemen and Syria. Thirdly, we did not consider further intersecting identities, for example race, ethnicity, religion of interviewees and how this might play out in leadership barriers for women. Fourthly, the individuals in FGDs do not reflect an equal number of men and women to demonstrate the diversity in understanding the role of women’s leadership in health and peacebuilding. Given our time limitations, we were unable to undertake further FGDs to complement this work. The findings from the FGDs were however useful in corroborating findings from the KIIs and applying these discussions in the context of an active conflict with significant influence from the donor community. While our findings can be viewed as narrow in the context of a range of conflict-affected settings, given the low number of individuals that contributed to this study, they offer a platform for advancing scholarship at this crucial nexus.

## Conclusion

This research demonstrates an instrumental link between health systems, women’s leadership, and peacebuilding. This nexus is essential for long-term stability, the right to health, and health system responsiveness. Health systems that are inclusive at the decision-making and leadership level enable all individuals to have voice and participate meaningfully within societies. However, our research supports the growing evidence that leaders in health systems are not gender diverse and demonstrates that women’s leadership may advance the invaluable connection between health systems and peacebuilding. Systems thinking across multiple sectors, like that of health and peace, ensure collaborative pursuits to dismantling the unequal power structures across many societies affected by conflict and give voice to the most marginalised [47, 54, 77]. Lederach’s framework therefore requires adaptation, as demonstrated by our findings, to ensure inter-sectoral dialogue in peacebuilding through the inclusion of both the health system and gender diversity at all leadership levels. The inclusion of women’s leadership is furthermore essential to the realisation of global goals, including the SDGs and the WPS agenda, that create equitable and peaceful societies.

## Supplementary Information

**Additional file 1.** Interview guide.

## Data Availability

The datasets generated and analysed during the current study are not publicly available due to them containing information that could compromise research participant privacy but are available from the corresponding author on reasonable request.

## Abbreviations

FGD: Focus group discussion
GBV: Gender based violence
GHPI: Global Health for Peace Initiative
HDP: Humanitarian-development-peace nexus
KII: Key informant interviews
MENA: Middle East and North Africa
NGO: Non-governmental organisation
SDG: Sustainable Development Goals
UN: United Nations
WHO: World Health Organisation
WPS: Women, Peace, and Security agenda
