# Supplementary material for: Strengthening health systems and peacebuilding through women’s leadership: a qualitative study
Source: Global Health. 2023 Mar 23;19:21. doi: 10.1186/s12992-023-00920-1 (PMC10035971; doi:10.1186/s12992-023-00920-1)
Supplement: Supplementary file 1 — Additional file 1. Interview guide. [file 12992_2023_920_MOESM1_ESM.docx]

**Interview guide**

1. What is your understanding of gender equality?
2. What is your understanding of “gender mainstreaming” (this can be in general and/or within your organisation)?
3. Building on your understanding of gender equality and gender mainstreaming:
   1. Has your organisation implemented any gender mainstreaming policies across all levels of your organisation?
   2. If such policies have been implemented, have they had an impact on your organisation’s culture and/or representation of women in leadership in your organisation?
   3. If such policies have not been implemented in your organisation specifically, have wider gender markers (for example, UN framework, national strategy) contributed to any change in organisational culture and/or representation of women in leadership in your organisation?
4. What is your understanding of the concept of leadership in both health systems and in peacebuilding?
5. How does your organisation support diverse leadership? Or how does it not support diverse leadership?
6. What do you perceive as barriers to women’s leadership (at the nexus of health, conflict, and peacebuilding)?
7. How can these barriers be overcome/mitigated?
8. What do you perceive as enablers to women’s leadership?
9. Can health be used as an entry point for peace building?
10. Are there any specific projects/programmes/interventions (current or potential) that you think the role of women’s leadership can strengthen the health and peacebuilding nexus
